# Preventive Effect of Probiotic *Leuconostoc mesenteroides* H40 Against Cognitive Disorder by Anti-Inflammatory, Synaptic Plasticity Regulation, and Antioxidant Effects

**DOI:** 10.3390/antiox14050565

**Published:** 2025-05-08

**Authors:** Na-Kyoung Lee, Yunjung Lee, Minhye Won, Nayeong Kim, Eunju Park, Hyun-Dong Paik

**Affiliations:** 1 Department of Food Science and Biotechnology of Animal Resources, Konkuk University, Seoul 05029, Republic of Korea; lnk11@konkuk.ac.kr; 2 Department of Food and Nutrition, Kyungnam University, Changwon 51767, Republic of Korea; hjlee@kyungnam.ac.kr (Y.L.); 2023220053@student.kyungnam.ac.kr (M.W.); kimnayoung99@student.kyungnam.ac.kr (N.K.); 3 View of Creativity, GHBio Co., Ltd., 120 Neungdong-Ro, Konkuk University, Seoul 05029, Republic of Korea

**Keywords:** probiotics, cognition, neurodegenerative disease, gut–brain axis, antioxidant

## Abstract

Live *Leuconostoc mesenteroides* H40 has been reported to have probiotic properties; however, the effect of its live probiotic form on cognitive ability has not been reported. This study investigated modulatory effects of the probiotic *L. mesenteroides* H40 in an ICR mouse model (male) of cognitive disorders. Cognitive disorders were induced in mice by the addition of scopolamine (1 mg/kg/day) with donepezil (2 mg/kg/day) as a medicinal control. *L. mesenteroides* H40 significantly attenuated scopolamine-induced cognitive disorder in the novel object recognition and Y-maze tests in a concentration-dependent manner. *L. mesenteroides* H40 decreased amyloid β levels, but increased β-secretase levels. The mRNA expression levels of *inducible nitric oxide synthase* (*iNOS*) and *cyclooxygenase (COX)-2* significantly decreased following *L. mesenteroides* H40 treatment. Additionally, TNF-α, IL-1β, and PGE2 protein expression was decreased. Acetylcholine, acetylcholinesterase, choline acetyltransferase, brain-derived neurotrophic factor (BDNF), and cAMP response element-binding protein (CREB) levels were increased in the brain tissues. The antioxidant effects of superoxide dismutase, catalase, and glutathione peroxidase were also alleviated. We demonstrated that *L. mesenteroides* H40 exhibits neuroprotective effects through anti-inflammatory, synaptic plasticity regulation, and antioxidant effects. Thus, the probiotic *L. mesenteroides* H40 could be used as a prophylactic functional food for cognitive disorders.

## 1. Introduction

Brain health is related to brain function, including the cognitive, sensory, social-emotional, behavioral, and motor domains. Conditions affecting the brain and nervous system typically occur throughout life and are characterized by impaired brain growth, damage to its structure, and/or impaired brain function. These include congenital and neurodevelopmental conditions and lifelong neurological disorders. Cognitive function is affected by aging, brain injury, environmental exposure to harmful pollutants (e.g., drugs or alcohol), and social interactions [1]. Cognitive impairment is a mental health disorder that affects learning, memory, perception, and problem-solving. Therefore, it has been associated with schizophrenia, depression, or dementia [2].

Scopolamine is a tropane alkaloid like hyoscyamine and atropine, both of which structurally copy the natural neurotransmitter acetylcholine [3]. As an acetylcholine analog, scopolamine antagonizes muscarinic acetylcholine receptors (mAChRs) throughout the central nervous system and body, causing various therapeutic and adverse effects related to changes in the parasympathetic nervous system and cholinergic signaling [3]. Scopolamine affects several behaviors, including anxiety, short-term memory, and acetylcholine levels.

Probiotics can improve the health of various organs, including gut, brain, and liver, via the gut axis [4,5]. The gut–brain axis is becoming a new strategy for brain health. Gut is a secondary brain because it produces mood-related substances, including serotonin, dopamine, and gamma-aminobutyric acid. The gut–brain axis is influenced by the cAMP response element-binding protein (CREB)/brain-derived neurotrophic factor (BDNF) signaling pathway [6]. Gut microbes are influenced by metabolites such as bile acids, dopamine (DA), γ-aminobutyric acid (GABA), norepinephrine (NE), serotonin (5-HT), histamine, short-chain fatty acids (SCFAs), etc. [4,5]. In addition, probiotics have been reported to affect SCFA production and metabolic activity (tryptophan metabolites, dopamine, trimethylamine oxide, and LPS) [4,5,6,7].

*Leuconostoc mesenteroides* has been used as a fermentation starter, and several *L. mesenteroides* strains have been used as probiotics for oral and brain health and longevity [8,9,10]. Additionally, extracellular vesicles from *L. mesenteroides* exhibit anti-inflammatory activity in microglia [11]. *L. mesenteroides* H40, a bacterial strain isolated from kimchi, exhibits probiotic properties in artificial gastric conditions [11]. Heat-killed *L. mesenteroides* H40 exhibited neuroprotective effects in SH-SY5Y cells (a neuroblastoma cell line) and cognitive effects in animal models [12,13]. However, live *L. mesenteroides* H40 has not yet been shown to affect cognitive function. Hence, this study aimed to investigate the preventative effects of live *L. mesenteroides* H40 on cognitive disorders in a scopolamine-treated mouse model.

## 2. Materials and Methods

### 2.1. Materials

*L. mesenteroides* H40 was manufactured and supplied by Mediogen (Jecheon, Republic of Korea), and freeze-dried *L. mesenteroides* H40 was used in the animal studies [12,13]. The samples were diluted and supplied using phosphate buffered saline (PBS) at a final concentration of 1 × 10^7^, 1 × 10^8^, or 1 × 10^9^ colony forming unit (CFU)/mice·day. Scopolamine was bought from Sigma-Aldrich (St. Louis, MO, USA).

### 2.2. Experimental Design for Examining the Cognitive Abilities

**Figure 1:**
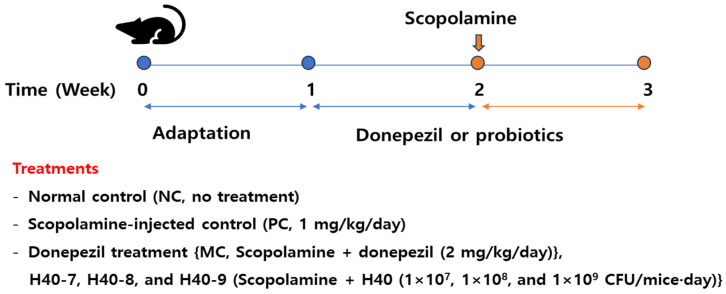
Schematic diagram for the experimental design.

The experimental plan and sample size performed as the guidelines of the Institutional Animal Care Committee of Kyungnam University (KUICA-24-03, approved on 9 July 2024). All 60 ICR male mice (7-week-old, 28–34 g) were purchased from Koatech (Seoul, Republic of Korea). To ensure consistency in the experimental outcomes, only male mice were used for all analyses. All mice were housed in groups two mice per cage (200 × 260 × 130 mm, polycarbonate) at 23 ± 2 °C, 51–55% relative humidity, and a 12 h light/dark cycle, and the cages were bedded with sawdust (Figure 1). After seven days of adaptation, the mice were randomly divided into six groups (10 mice per group) based on their body weight as follows: normal control (NC, no treatment), scopolamine-injected control (PC, 1 mg/kg/day), donepezil treatment {MC, treated with scopolamine (1 mg/kg/day) and donepezil (2 mg/kg/day)}, and H40 treatments {H40-7, H40-8, and H40-9 (treated with scopolamine (1 mg/kg/day) and H40 treatment (1 × 10^7^, 1 × 10^8^, and 1 × 10^9^ CFU/mice·day)}, with four replicates per treatment. The sample size (*n* = 10 per group) was determined based on an a priori power analysis (α = 0.05, power = 0.80) using one-way analysis of variance (ANOVA). To induce cognitive disorder, scopolamine was injected intraperitoneally on days 7–14, and the samples or donepezil were administered for two weeks. No other adverse reactions were observed during administration. The animals were not fed for 12 h and euthanized under anesthesia induced by isoflurane (4 mL/kg) after 2 weeks of treatment. Brain and hippocampal tissues were separated on ice and frozen at −80 °C for further analysis. Brain tissue from six or four mice were randomly selected for RT-PCR or western blot analysis, respectively. Hippocampal tissues from each mouse were bisected and randomly divided into five samples for the analysis of amyloid β accumulation, cytokines, choline-based substances, and antioxidant enzyme activity.

### 2.3. Novel Object Recognition and Y-Maze Test

To assess object recognition ability, the novel object recognition and Y-maze tests were performed using behavioral tests [5]. For the novel object recognition test, the mice were placed in a box with two comparable objects (O1 + O2) and allowed to adapt for 3 min. The stay time and number of times on each object were recorded over 5 min. The following day, O1 was replaced with O2. The mice were given 5 min to explore the novel objects, and the time spent was used to determine the recognition index. The number of touches on a new object indicated the cognitive recognition of that object.

The Y-maze test used an apparatus with three arms (50 cm × 20 cm × 10 cm; length × height × width) [5]. The arms set approximately 10 cm from the free ends. All mice underwent two training trials per day for two consecutive days. Each mouse was placed at the end of one arm and allowed to move freely through the maze for 8 min. During the trial assemblies, the mice were placed at the center of the apparatus and allowed to move freely for 8 min to explore their arms at random. The experimental assemblies were conducted over four days. The total number of entries into each arm was recorded during each session. Spontaneous alternation of the mice was determined.

The apparatus used for the novel object recognition test and the maze arms were thoroughly cleaned between tasks to remove residual odors. All behavioral experiments were carried out in a room adjacent to that in which the mice were housed under the same conditions of temperature and humidity and light cycle. Animals were sacrificed one day after the behavioral analysis.

### 2.4. β-Secretase, Amyloid β, Acetylcholine, Acetylcholinesterase, and Choline Acetyltransferase

β-secretase levels in the supernatants were measured using a mouse Bace1 enzyme-linked immunosorbent assay (ELISA) kit (Express Biotech International, Frederick, MD, USA). The hippocampal tissue was homogenized in ice-cold phosphate-buffered saline (pH 7.4) and centrifuged at 5000× *g* for 5 min at 4 °C. The supernatant was used to determine the β-secretase level. Aβ40 and Aβ42 levels in hippocampal tissue were measured using mouse amyloid β40 and β42 ELISA kit (BioVendor R&D, Brno, Czech Republic). First, the hippocampal tissue was homogenized on ice in 5 M guanidine-HCl/50 mM Tris buffer (8×, pH 8.0). The hippocampal homogenate was incubated at about 23 °C for 3–4 h, mixed with cold PBS (10×), and centrifuged at 16,000× *g* for 20 min to obtain the supernatant. The Aβ40 and Aβ42 levels in the supernatant were calculated according to the manufacturer’s instructions. Equal amounts of all samples were analyzed after the protein assay using the Micro BCA protein assay kit (Thermo Fisher Scientific, Rockford, IL, USA).

To obtain acetylcholine (ACh), acetylcholinesterase (AChE), and choline acetyltransferase (ChAT) concentrations, hippocampal tissues were mixed with cold 0.1 M phosphate-buffered saline (pH 7.5) and centrifuged (10,000× *g* for 5 min) to obtain the supernatants. The supernatants were analyzed according to the instructions of BioAssay Systems (Hayward, CA, USA) and MyBioSource Inc. (San Diego, CA, USA). Equal amounts of all samples were analyzed after the protein assay.

### 2.5. Cytokines and Factors Related to Neuroinflammatory Effect

The mRNA expression in the brain tissue was determined using TRIzol reagent (Invitrogen, Carlsbad, CA, USA), M-MLV reverse transcriptase (Promega, Solana Beach, CA, USA), and real-time PCR. The used sequences of primers were as follows: iNOS, 5′-ctatggccgctttgatgtgc-3′ and 5′-tggggatgctccatggtcac-3; COX-2, 5′-acctggtgaactacgactgc-3′ and 5′-ctagggaggggactgctcat-3′. The reference gene used was a β-actin encoding gene. The normalized target gene expression levels in each sample were calculated using 2^−∆∆*C*T^. The values were stated as fold compared to the control.

After normalization of the hippocampal tissue, the cells were immediately centrifuged (10,000× *g*, 5 min) to obtain the supernatants. The hippocampal tissue was homogenized in ice-cold Tissue Protein Extraction Reagent (Thermo Fisher Scientific) and centrifuged at 10,000× *g* for 15 min at 4 °C. The supernatant was used to determine the TNF-α, IL-1β, and PGE2 level, and the protein concentrations of the supernatants were determined. TNF-α and IL-1β were determined using an ELISA kit obtained from BD (San Diego, CA, USA), and PGE2 was analyzed using an ELISA kit purchased from MyBioSource (San Diego, CA, USA). The concentrations of TNF-α, IL-1β, and PGE2 were determined at 450 nm using an ELISA reader (Thermo Fisher Scientific).

### 2.6. BDNF and CREB Production in Brain Tissue

The sequences of the sense and antisense primers used for amplification were as follows: β-actin, sense, 5′-ttccagccttccttcttg-3′ and antisense, 3′-ggagccagagcagtaatc-5′; BDNF, sense, 5′-actgcagtggacatgtctgg-3′ and antisense, 5′-ctgcagccttccttggtgta-3′; CREB, sense, 5′-actcagccgggtactaccat-3′ and antisense, 5′-acgccataacaactccaggg-3. The β-actin gene was used as the reference gene. The gene expression levels were calculated using 2^−∆∆CT^ in each sample.

For protein analysis, hippocampal tissue was organized using ice-cold homogenization buffer (50 mM Tris, 0.25% sodium dodecyl sulfate (SDS), 150 mM NaCl, 1% NP-40, and 1 mM ethylenediaminetetraacetic acid (EDTA), pH 7.4) and centrifuged (10,000× *g*, 15 min at 4 °C). After quantification, 20 μg of protein from each sample was resolved by SDS-PAGE. Proteins were transferred onto nitrocellulose membranes using Trans-Blot^®^ SD Semi-Dry Transfer Cell (Bio-Rad, Hercules, CO, USA). Staining was performed using Amersham high-range molecular weight rainbow markers. The nitrocellulose membranes were washed with distilled water and blocked with blocking buffer for 2 h. The membranes were then incubated for 12 h at 4 °C with antibodies specific to BDNF and CREB (Novus Biologicals, Centennial, CO, USA). The next day, the membranes were washed with TBST and incubated with the appropriate secondary antibodies for 2 h at room temperature. The membranes were washed with TBST for 30 min. The protein bands were visualized by enhanced chemiluminescence using an enhanced chemiluminescence kit. Band densities were measured using Image Lab (Bio-Rad, Boulder, CO, USA). Equal amounts of all samples were analyzed after the protein assay.

### 2.7. Antioxidant Activity

The hippocampal tissue was mixed in cold 20 mM HEPES buffer (pH 7.2) containing 210 mM mannitol, 1 mM EGTA, and 70 mM sucrose. The resulting hippocampal homogenate was centrifuged to obtain the supernatant (1500× *g* for 5 min). Superoxide dismutase (SOD) activity in the supernatant was measured using an ELISA kit (Cayman Chemical, Ann Arbor, MI, USA).

To determine the activity of catalase and glutathione peroxidase (GSH-Px), hippocampal tissues were homogenized and centrifuged at 10,000× *g* for 15 min to obtain the supernatant. Catalase and GSH-Px levels in hippocampal tissue were measured using an ELISA kit (Cayman Chemical, Ann Arbor, MI, USA).

### 2.8. Statistical Evaluation

Used statistical methods were one-way ANOVA, Duncan’s multiple range test, and *t*-test for multiple comparisons and represented as the mean ± standard error of three replicates. Statistical analysis was performed using SPSS software (SPSS Inc., Chicago, IL, USA, version 20.0), and the results were regard as statistically significant at *p* < 0.05.

## 3. Results

### 3.1. Effect of Probiotic H40 on Recognition Index, Object Cognitive Ability, and Y-Maze Tests

**Figure 2:**
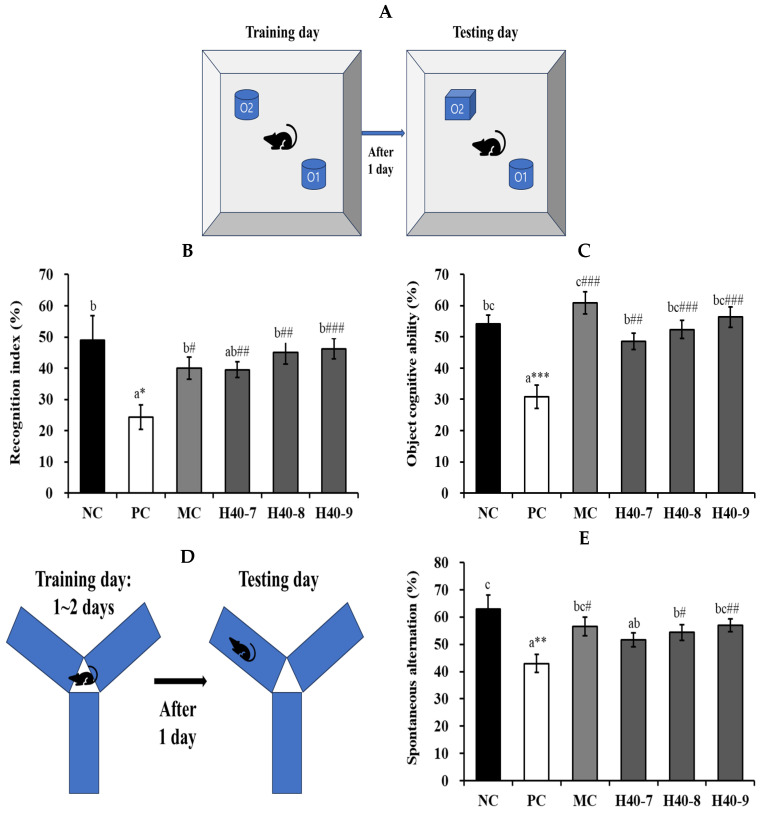
Effects of probiotic *Leuconostoc mesenteroides* H40 on behavioral tests using the novel object exploration and Y-maze test in mice with scopolamine-induced memory disorder (*n* = 10). (**A**) Scheme of novel object exploration, (**B**) recognition index (%), (**C**) object cognition ability (%), (**D**) scheme of Y-maze, and (**E**) spontaneous alternation (%). Data are presented as the mean ± standard error, and alphabetic letters on the error bars indicate significant differences (*p* < 0.05). NC, normal control without scopolamine; PC, positive control with scopolamine; DO, PC with 2 mg/kg donepezil; H40-7, PC with 1 × 10^7^ CFU/mice·day H40; H40-8, PC with 1 × 10^8^ CFU/mice·day H40; H40-9, PC with 1 × 10^9^ CFU/mice·day H40. * *p* < 0.05, ** *p* < 0.01, *** *p* < 0.001 compared with the NC group. ^#^ *p* < 0.05, ^##^ *p* < 0.01, ^###^ *p* < 0.001 compared with the PC group.

When recognition indices and object cognitive abilities were tested using novel objects, changes were observed in mice administered scopolamine (Figure 2A–C). In the cognitively deficit group (scopolamine-treated control, PC), the recognition index was 24.33 ± 3.87% (Figure 2B). However, the NC group, which was not administered scopolamine, showed 48.96 ± 7.86%, and the recognition index for novel objects significantly increased during the stay time compared with the PC group (*p* < 0.05). The proportions of the H40-7, H40-8, and H40-9 groups were 39.52 ± 2.57% (*p* < 0.01), 45.04 ± 3.73% (*p* < 0.01), and 46.27 ± 3.21% (*p* < 0.001), respectively. Additionally, the MC group had a recognition index of 40.03 ± 3.56%. In object cognitive ability, the PC group (30.79 ± 3.77%, *p* < 0.001) was found to have significantly lower object cognitive ability than the NC group (54.22 ± 2.85%) (Figure 2C). The H40-7, H40-8, and H40-9 groups showed 48.64 ± 2.61% (*p* < 0.01), 52.39 ± 2.92% (*p* < 0.001), and 56.36 ± 3.25% (*p* < 0.001), respectively. The MC group (60.89 ± 3.54%, *p* < 0.001) showed a significantly increased object cognitive ability compared to the PC group.

The Y-maze test revealed a clear pattern of spontaneous alterations in the tested groups (Figure 2D,E). The PC group (43.01 ± 3.36%, *p* < 0.01) showed significantly less alternation behavior than did the NC group (62.93 ± 5.05%), indicating that cognitive dysfunction was induced. Additionally, spontaneous alteration was significantly increased in the H40-8 (54.35 ± 2.82%, *p* < 0.05), H40-9 (56.95 ± 2.29%, *p* < 0.01), and MC (56.54 ± 3.48%, *p* < 0.05) groups compared to that in the PC group, indicating an improvement in cognitive ability. The performance in the H40-9 group was similar to that in the MC group.

### 3.2. Effect of Probiotic H40 on Amyloid b40, Amyloid b42, and b-Secretase in Hippocampal Tissue

**Figure 3:**
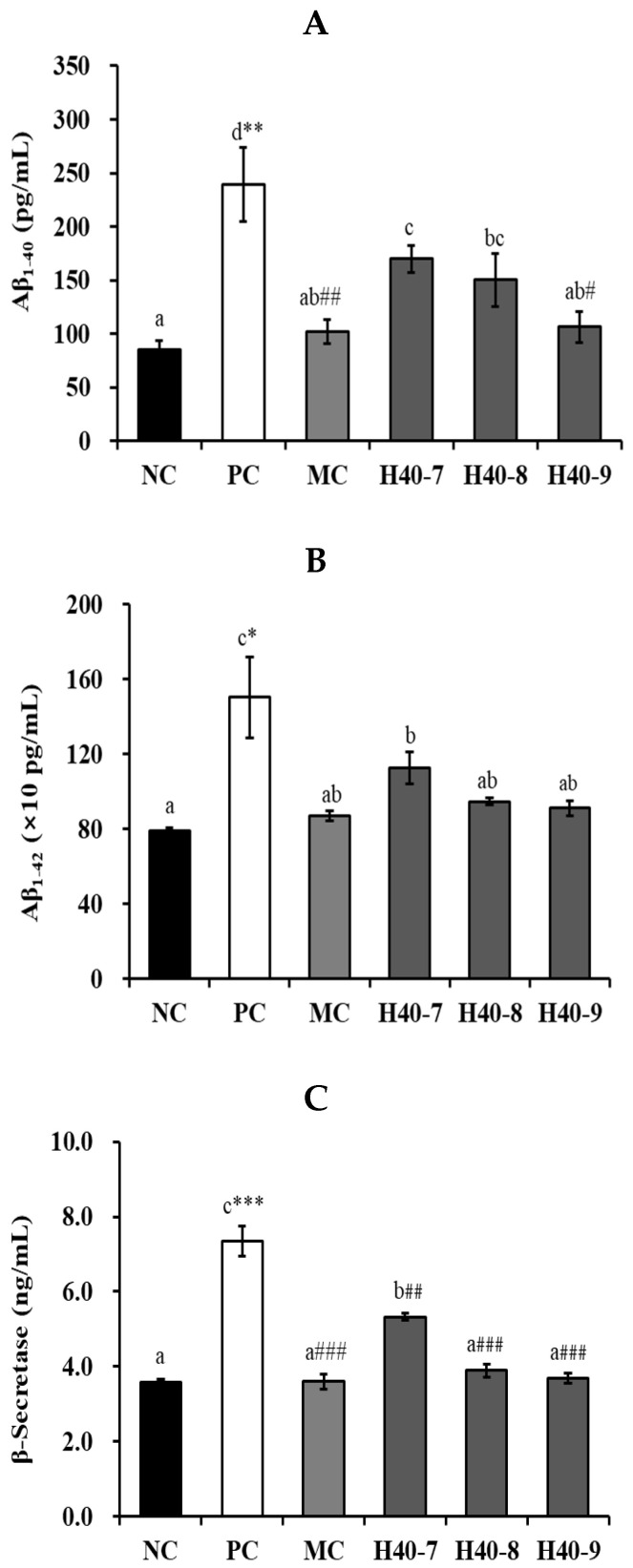
Effects of probiotic *Leuconostoc mesenteroides* H40 on amyloid β accumulation in the hippocampal tissue using an ELISA kit. (**A**) Amyloid β40, (**B**) amyloid β42, and (**C**) β-secretase activities. Data are presented as the mean ± standard error, and different letters on error bars indicate significant differences (*n* = 5, *p* < 0.05). NC, normal control without scopolamine; PC, positive control with scopolamine; DO, PC with 2 mg/kg donepezil; H40-7, PC with 1 × 10^7^ CFU/mice·day H40; H40-8, PC with 1 × 10^8^ CFU/mice·day H40; H40-9, PC with 1 × 10^9^ CFU/mice·day H40. * *p* < 0.05, ** *p* < 0.01, *** *p* < 0.001 compared with the NC group. ^#^ *p* < 0.05, ^##^ *p* < 0.01, ^###^ *p* < 0.001 compared with the PC group.

The Aβ40 content of the PC group (239.32 ± 34.84 pg/mL, *p* < 0.01) significantly increased compared to that in the NC group (84.95 ± 9.10 pg/mL) (Figure 3A), and the corresponding values of the H40-7, H40-8, H40-9, and MC groups were 169.83 ± 12.66, 150.22 ± 25.08, 106.51 ± 14.54, and 101.93 ± 11.53 pg/mL, respectively, with those of the H40-9 (*p* < 0.05) and MC (*p* < 0.01) groups showing a significant decrease compared to the PC group. Additionally, the Aβ42 content was significantly increased in the PC group (1502.88 ± 217.30 pg/mL, *p* < 0.05) compared to the values in NC group (790.73 ± 17.25 pg/mL) (Figure 3B), and was 1126.61 ± 85.06, 946.46 ± 19.36, 910.62 ± 38.62, and 870.75 ± 26.48 pg/mL in the H40-7, H40-8, H40-9, and MC groups, respectively. These results show that Aβ accumulation was slightly reduced.

The PC group (7.35 ± 0.40 ng/mL, *p* < 0.001) showed a significantly increased β-secretase levels compared to those the NC group (3.59 ± 0.08 ng/mL) (Figure 3C). The H40-7 (5.33 ± 0.10 ng/mL, *p* < 0.01), H40-8 (3.90 ± 0.18 ng/mL, *p* < 0.001), H40-9 (3.69 ± 0.12 ng/mL, *p* < 0.001), and MC (3.60 ± 0.20 ng/mL, *p* < 0.001) groups showed a significantly decreased β-secretase level in contrast to the PC group.

### 3.3. Effect of Probiotic H40 on Anti-Neuroinflammatory Effect in Hippocampal Tissue

**Figure 4:**
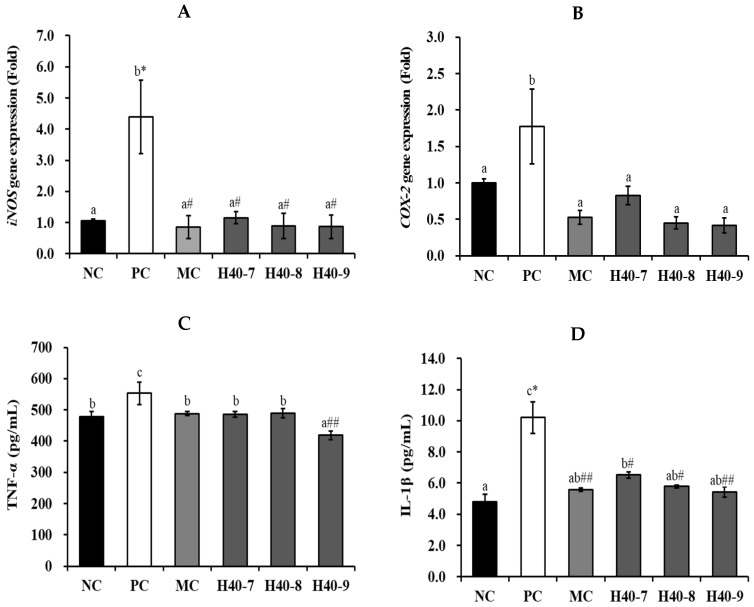

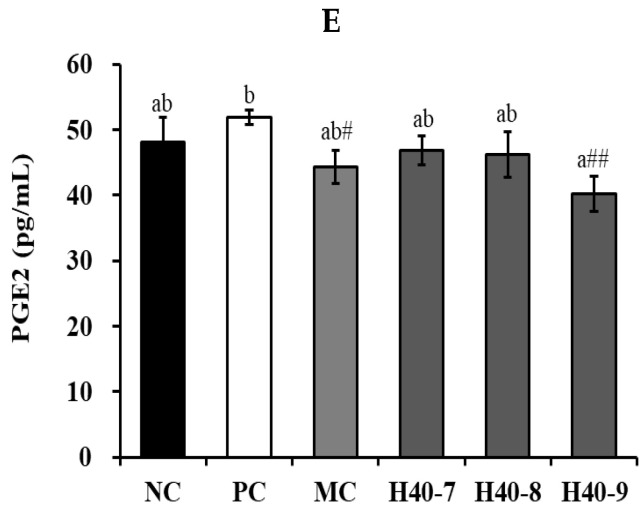
The anti-neuroinflammatory effect of probiotic *Leuconostoc mesenteroides* H40 on hippocampal proteins. (**A**) *iNOS* and (**B**) *COX-2* expression levels in brain tissue using rt-PCR. (**C**) TNF-α, (**D**) IL-1β, and (**E**) PGE2 expression levels in hippocampal tissue using ELISA kit. Each value are presented as the mean ± standard error, and different letters on the error bars indicate significant differences (*n* = 5, *p* < 0.05). NC, normal control without scopolamine; PC, positive control with scopolamine; DO, PC with 2 mg/kg donepezil; H40-7, PC with 1 × 10^7^ CFU/mice·day H40; H40-8, PC with 1 × 10^8^ CFU/mice·day H40; H40-9, PC with 1 × 10^9^ CFU/mice·day H40. * *p* < 0.05 compared with the NC group. ^#^ *p* < 0.05 and ^##^ *p* < 0.01 compared with the PC group.

*iNOS* and *COX-2* mRNA expression levels were determined by RT-PCR (Figure 4A,B). In the case of *iNOS*, the PC group (4.40 ± 1.18-fold, *p* < 0.05) showed a significant increase compared with the NC group (1.06 ± 0.05-fold). However, the H40-7, H40-8, H40-9, and MC groups exhibited 1.16 ± 0.20-, 0.89 ± 0.41-, 0.87 ± 0.39-, and 0.86 ± 0.37-fold lower expression levels, respectively (*p* < 0.05). The PC group (1.77 ± 0.51-fold) showed a increment in *COX-2* expression in the hippocampal tissue compared to that in the NC group (1.00 ± 0.05-fold), indicating that brain tissue cells were damaged after scopolamine treatment. The H40-7, H40-8, H40-9, and MC groups exhibited lower expression levels (0.83 ± 0.13-, 0.45 ± 0.08-, 0.42 ± 0.10-, and 0.53 ± 0.09-fold, respectively).

TNF-α, IL-1β, and PGE2 protein expression levels were assessed (Figure 4C–E). The PC group (533.30 ± 35.75 pg/mL) increased TNF-α expression compared with the NC group (477.82 ± 17.32 pg/mL), whereas the values in the H40-7, H40-8, H40-9, and MC group were 485.90 ± 9.70, 490.14 ± 14.67, 418.64 ± 13.53, and 488.31 ± 7.04 pg/mL, respectively. The PC group (10.20 ± 1.01 pg/mL, *p* < 0.05) also showed significantly increased IL-1β expression compared to that in the NC group (4.80 ± 0.47 pg/mL). The IL-1β expression levels for the H40-7, H40-8, H40-9, and MC groups were 6.52 ± 0.20 (*p* < 0.05), 5.78 ± 0.10 (*p* < 0.05), 5.41 ± 0.34 (*p* < 0.01), and 5.58 ± 0.12 pg/mL (*p* < 0.01), respectively. Notably that the H40 groups showed concentration-dependent results. The PC group (51.86 ± 1.14 pg/mL) also showed increased PGE2 production in comparison to the NC group (48.04 ± 3.90 pg/mL). The corresponding values for the H40-7, H40-8, H40-9, and MC groups were 46.84 ± 2.19, 46.27 ± 3.46, 40.23 ± 2.72 (*p* < 0.01), and 44.30 ± 2.54 (*p* < 0.05) pg/mL, respectively. Notably, the H40 group showed concentration-dependent results.

### 3.4. Effect of Probiotic H40 on Acetylcholine, Acetylcholine Esterase, and Choline Acetyltransferase in Hippocampal Tissue

**Figure 5:**
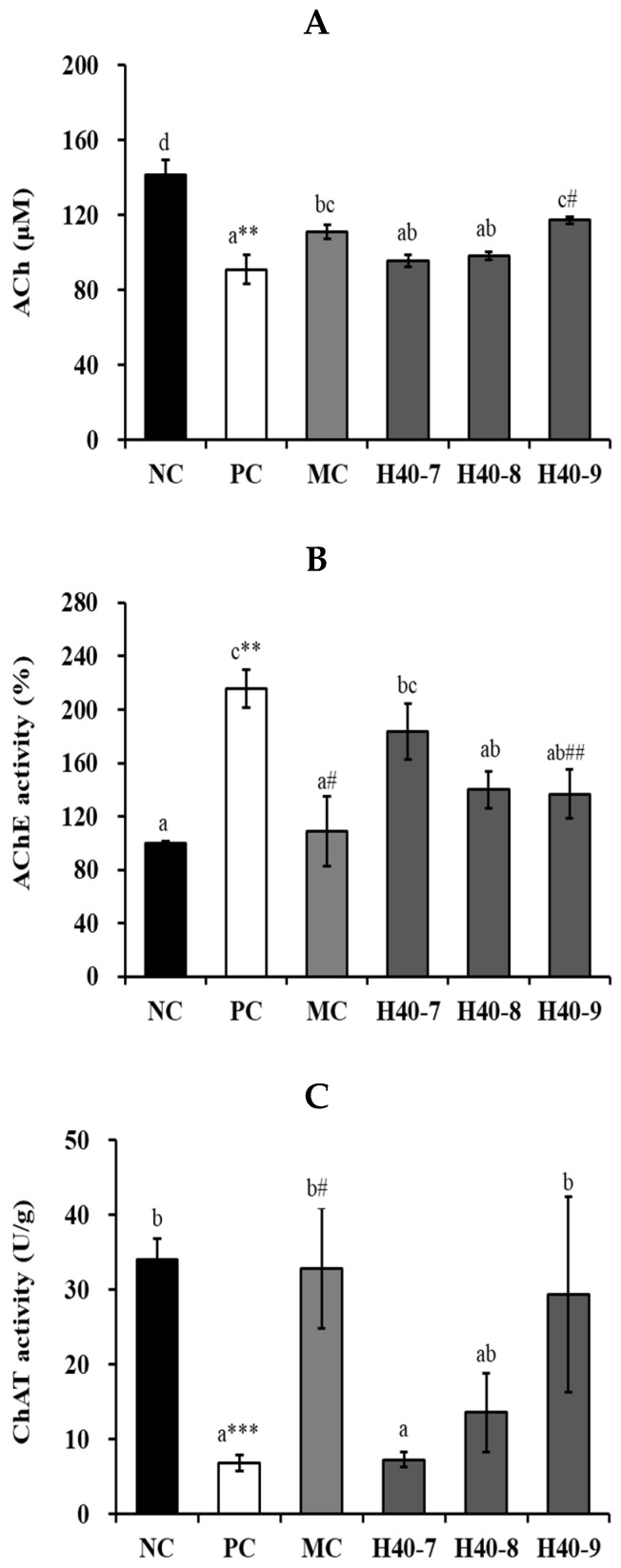
Effects of probiotic *Leuconostoc mesenteroides* H40 on choline-based substances in hippocampal tissue, assessed by ELISA. Production of (**A**) ACh, (**B**) AChE, and (**C**) ChAT. Data are presented as the mean ± standard error and alphabetic letters on the error bars indicate significant differences (*n* = 5, *p* < 0.05). NC, normal control without scopolamine; PC, positive control with scopolamine; DO, PC with 2 mg/kg donepezil; H40-7, PC with 1 × 10^7^ CFU/mice·day H40; H40-8, PC with 1 × 10^8^ CFU/mice·day H40; H40-9, PC with 1 × 10^9^ CFU/mice·day H40. ** *p* < 0.01, *** *p* < 0.001 compared with the NC group. ^#^ *p* < 0.05 and ^##^ *p* < 0.01 compared with the PC group.

ACh content was significantly decreased in the PC group (90.90 ± 7.68 μM, *p* < 0.01) in comparison to that in the NC group (141.60 ± 7.75 μM) (Figure 5A). The H40-7 (95.64 ± 3.36 μM), H40-8 (98.32 ± 2.23 μM), H40-9 (117.30 ± 1.94 μM), and MC (111.21 ± 3.83 μM) groups showed an increase in ACh, and the H40-9 group showed significant differences compared to the values in the PC group (*p* < 0.05).

Figure 5B shows that the PC group (215.35 ± 14.25%, *p* < 0.01) showed significantly higher AChE activity than the NC group (100.00 ± 1.21%). The H40-9 (136.49 ± 18.30%, *p* < 0.01), and MC (108.79 ± 25.81%, *p* < 0.05) groups showed significantly lower AChE activities than the PC group, indicating cognitive impairment.

The PC group (6.81 ± 1.04 U/g, *p* < 0.001) showed a significant decrement in ChAT activity in comparison to that in NC group (34.08 ± 2.74 U/g), whereas the H40-9 (29.32 ± 13.07 U/g) group showed a significant increment in ChAT activity compared with the PC group (Figure 5C).

### 3.5. Effect of Probiotic H40 on BDNF and CREB Expression in Hippocampal Tissue

**Figure 6:**
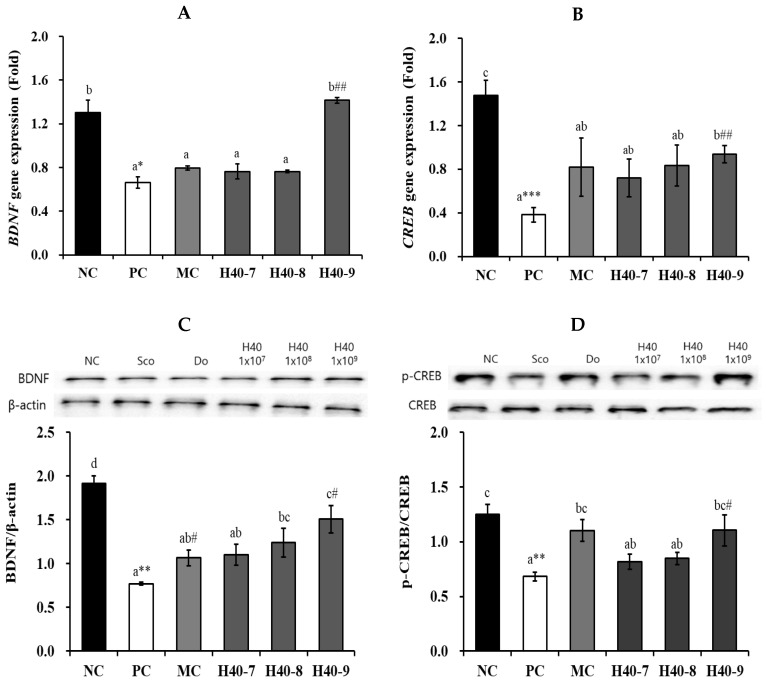
Effects of probiotic *Leuconostoc mesenteroides* H40 on BDNF and CREP production. Gene expression of (**A**) *BDNF* and (**B**) *CREB* using rt-PCR. (**C**) BDNF/β-actin ratio and (**D**) p-CREB/CREB ratio using western blotting. Data are presented as the mean ± standard error. Different letters on the error bars indicate significant differences (*n* = 5, *p* < 0.05). NC, normal control without scopolamine; PC, positive control with scopolamine; DO, PC with 2 mg/kg donepezil; H40-7, PC with 1 × 10^7^ CFU/mice·day H40; H40-8, PC with 1 × 10^8^ CFU/mice·day H40; H40-9, PC with 1 × 10^9^ CFU/mice·day H40. * *p* < 0.05, ** *p* < 0.01, *** *p* < 0.001 compared with the NC group. ^#^ *p* < 0.05 and ^##^ *p* < 0.01 compared with the PC group.

*BDNF* mRNA expression levels varied in the hippocampi of the mice (Figure 6A). The PC group (0.66 ± 0.05-fold, *p* < 0.05) showed a significant decrease in *BDNF* mRNA expression in comparison to values in the NC group (1.30 ± 0.11-fold). There was no change in *BDNF* mRNA expression in the H40-7 (0.76 ± 0.01-fold) and H40-8 (0.76 ± 0.01-fold) groups, but *BDNF* mRNA expression was significantly increased in the H40-9 group (1.42 ± 0.03-fold, *p* < 0.01) compared to that in the PC group. Additionally, the H40-9 group showed *BDNF* mRNA expression at the same level as that in the NC group.

The PC group (0.38 ± 0.07-fold, *p* < 0.001) showed a significant decrement in *CREB* mRNA expression compared to that in the NC group (1.48 ± 0.14-fold) (Figure 6B). In the H40-7 and H40-8 groups, *CREB* mRNA expression increased 0.72 ± 0.17-fold (88.4%) and 0.83 ± 0.19-fold (118.4%), respectively, comparison to the values in the PC group, but the difference was not statistically significant. In the H40-9 group, *CREB* mRNA expression was significantly increased (0.94 ± 0.08-fold, 145.6%, *p* < 0.01) compared to that in the PC group. The MC group comprised 0.82 ± 0.27-fold (114.3%) of the patients.

The BDNF/β-actin ratio was significantly reduced in PC group (0.77 ± 0.02-fold, *p* < 0.01) compared to NC group (1.91 ± 0.09-fold) (Figure 6C). The BDNF/β-actin ratio was increased in the H40-7 (1.10 ± 0.12-fold, 43.2%) and H40-8 (1.24 ± 0.16-fold, 60.9%) groups compared to the PC group, the differences were not statistically significant. In contrast, the H40-9 (1.51 ± 0.16-fold, 95.5%, *p* < 0.01) and MC (1.07 ± 1.07-fold, 38.3% *p* < 0.01) groups showed a significant increase in the BDNF/β-actin ratio relative to the PC group.

The p-CREB/CREB ratio was significantly reduced in PC (0.68 ± 0.04-fold, *p* < 0.01) compared to NC group (1.25 ± 0.09-fold) (Figure 6D). In the H40-7 and H40-8 groups, the p-CREB/CREB ratio increased 0.82 ± 0.07-fold (19.5%) and 0.85 ± 0.06-fold (24.2%), respectively, compared to that in the PC group; however, the difference was not statistically significant. Additionally, the H40-9 group showed a significant increase of 1.10 ± 0.14-fold (61.7%, *p* < 0.05), similar to MC (1.10 ± 0.10-fold, 61.5%, *p* < 0.05).

### 3.6. Antioxidant Effect of Probiotic H40 in Hippocampal Tissue

**Figure 7:**
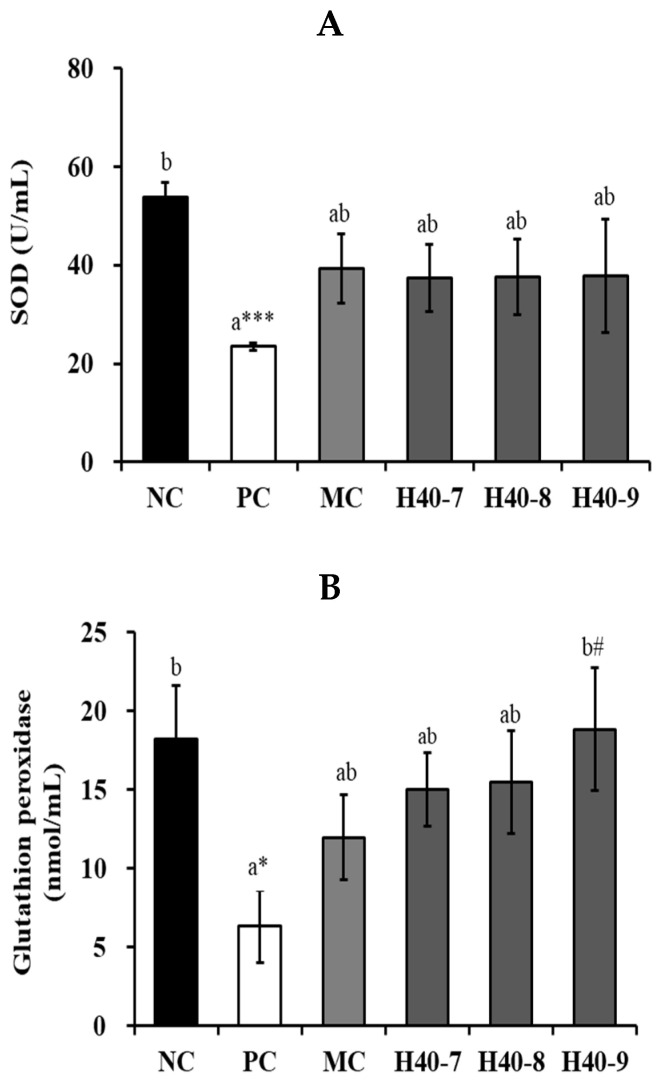

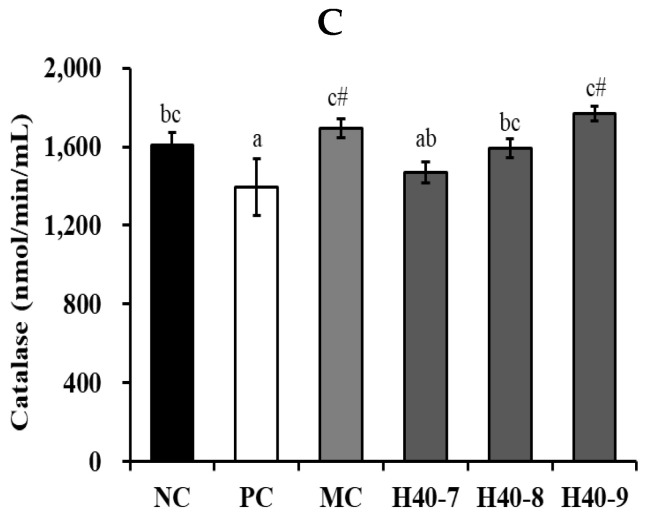
Antioxidant effects of probiotic *Leuconostoc mesenteroides* H40 in hippocampal tissue using ELISA kit. Activity of (**A**) super oxide dismutase, (**B**) glutathione peroxidase, and (**C**) catalase. Data are presented as the mean ± standard error and alphabetical letters on the error bars indicate significant differences (*n* = 5, *p* < 0.05). NC, normal control without scopolamine; PC, positive control with scopolamine; DO, PC with 2 mg/kg donepezil; H40-7, PC with 1 × 10^7^ CFU/mice·day H40; H40-8, PC with 1 × 10^8^ CFU/mice·day H40; H40-9, PC with 1 × 10^9^ CFU/mice·day H40. * *p* < 0.05, *** *p* < 0.001 compared with the NC group. ^#^ *p* < 0.05 compared with the PC group.

The antioxidant effects of H40 were confirmed based on the production of superoxide dismutase (SOD), GSH-Px, and catalase (Figure 7). In SOD activity, the PC group (23.48 ± 0.74 U/mL, *p* < 0.001) showed a significant decrement in the hippocampal tissue compared to that in the NC group (53.82 ± 3.03 U/mL) (Figure 7A). In the H40-7, H40-8, and H40-9 groups, SOD activity increased by 37.43 ± 6.78 U/mL (59.4%), 37.62 ± 7.73 U/mL (60.2%), and 37.84 ± 11.47 U/mL (61.2%), respectively, compared to that in PC group, but there was no statistical significance. The MC group showed 39.33 ± 7.01 U/mL (67.5%).

The PC group (6.31 ± 2.28 nmol/min/mL, *p* < 0.05) showed significantly decreased GSH-Px activity compared with that in the NC group (18.24 ± 3.37 nmol/min/mL) (Figure 7B). In the H40-7 and H40-8 groups, GSH-Px activity increased by 15.03 ± 2.34 nmol/min/mL (138.2%) and 15.46 ± 3.27 nmol/min/mL (145.0%), respectively, compared to that in the PC group, but there was no statistical significance. In the H40-9 group, GSH-Px activity significantly increased by 18.84 ± 3.91 nmol/min/mL (198.6%, *p* < 0.05) compared to that in the PC group. Additionally, the H40-9 group showed GSH-Px activity similar to that in the NC group. MC group demonstrated 11.95 ± 2.71 nmol/min/mL (89.4%), although the difference was not statistically significant.

The PC group (1392.93 ± 144.90 nmol/min/mL) showed a significant decrease in catalase activity compared to that in NC (1609.11 ± 64.16 nmol/min/mL) (Figure 7C). In contrast, the H40-9 (1768.52 ± 35.33 nmol/min/mL, 27.0%, *p* < 0.05) and MC (1692.82 ± 46.34 nmol/min/mL, 21.5%, *p* < 0.05) groups showed a significant increase in catalase activity compared to the PC group. Additionally, the H40-8 and H40-9 groups showed catalase activities similar to those in the NC group. MC group demonstrated 1692.82 ± 46.34 nmol/min/mL (21.5%, *p* < 0.05).

## 4. Discussion

This study investigated the cognitive-attenuating effects of live *L. mesenteroides* H40 in a mouse model of scopolamine-induced cognitive disorders. Probiotic *L. mesenteroides* H40 treatment showed enhanced learning and memory abilities and suppressed neuroinflammation and Aβ accumulation. Additionally, probiotic *L. mesenteroides* H40 treatment increased the neurotransmitter and antioxidant effects.

Probiotics and paraprobiotics are live and dead microorganisms, respectively, that provide health benefits when administered in adequate amounts [14]. The difference between probiotics and paraprobiotics is the type of microorganisms used in them, i.e., living (probiotics) or non-living (paraprobiotics), and their effective concentrations. Additionally, probiotics can be metabolized in the intestine and affect gut microbiota. However, the cellular composition of the living or dead cells remained unchanged. Therefore, the health functions of these substances may vary depending on their metabolic capacity and effective concentration in the intestine. Lee et al. [13] reported the cognition-alleviating effects of the paraprobiotic *L. mesenteroides* H40 administered daily at 8–9 log CFU/mouse. However, this study investigated the cognition-alleviating effects of the live probiotic *L. mesenteroides* H40 at 7–9 log CFU/mouse. Overall, the probiotic *L. mesenteroides* H40 had a greater alleviating effect than that of parabiotic H40. The effect of the probiotic *L. mesenteroides* H40 at 10^8^ CFU/mice·day was similar to that of the parabiotic H40 at 10^9^ cells/mice·day. Rezaie et al. [15] have reported that comparison of probiotics and paraprobiotics has similar results in dextran sodium sulfate (DSS)-induced colitis. This study suggests that the selection of probiotics or paraprobiotics depends on the health status and needs of consumers, considering their safety and efficacy.

In mouse models, scopolamine induces cognitive deficits by promoting neuroinflammation and oxidative stress [16]. Cholinergic neurons in the central nervous system (CNS) reduce in an approach associated with cognitive loss in patients with AD and senile dementia [17]. Figure 2 shows behavioral changes induced by scopolamine treatment. In this study, the scopolamine-treated PC group showed cognitive decline and spontaneous alteration, whereas treatment with live *L. mesenteroides* H40 or donepezil ameliorated these effects.

β-Secretase inhibitors have been considered as preventive treatments for AD because β-secretase increases the accumulation of Aβ and Aβ plaques [18,19]. Our results showed that probiotic *L. mesenteroides* H40 could reduce Aβ and β-secretase production in scopolamine-treated mice in a manner similar to that treated with donepezil (Figure 3). Similarly, another study showed that heat-killed *Lactococcus lactis* KC24 and probiotic *Bifidobacterium breve* MCC1274 inhibited β-secretase and Aβ production [5,20].

As neuroinflammation and aging can accelerate neurodegeneration, anti-inflammatory and antioxidant effects are targets for addressing brain health [21]. TNF-α and IL-1β are directly associated with brain function and can advance inflammatory response inside the brain [22,23,24]. As shown in Figure 4, probiotic *L. mesenteroides* H40 showed a protective effect against neuroinflammation by dose-dependently reducing TNF-α, IL-1β, iNOS, and COX-2 levels. PGE2 is a brain lipid signaling molecule taken from the cell membrane through the activity of COX-1 and COX-2 [25]. PGE2 regulates NO synthase, induces cell migration, and promotes neuroproliferation, thus exerting a neuroprotective role [26]. Therefore, PGE2 levels may affect cognition, learning, and neuroinflammatory responses in the brain. Our results showed that PC increased PGE2 levels, whereas the probiotics *L. mesenteroides* H40 and donepezil decreased them similar to NC.

Synaptic plasticity is connected with ACh, AChE, and ChAT activities. Figure 5 showed that scopolamine treatment decreased ACh, AChE, and ChAT activities. ACh shows an important role in the peripheral and central nervous systems, whereas AChE hydrolyzes ACh in the synaptic cleft. ChAT is accountable for acetylcholine synthesis and is the most principal marker of the functional status of cholinergic neurons [27]. Cholinergic neurons are placed in the basal forebrain, containing neurons that creäte the nucleus basalis of Meynert cells in AD [28]. AChE was increased by scopolamine, and the probiotic *L. mesenteroides* H40 at 10^8^ and 10^9^ CFU/mice·day decreased, similar to donepezil. However, scopolamine decreased ACh and ChAT levels. The probiotic *L. mesenteroides* H40 at 10^9^ CFU/mice·day and donepezil treatment increased these values.

BDNF is involved in neuronal survival, migration, differentiation, axonal and dendritic outgrowth, synaptogenesis, synaptic plasticity, behavioral regulation, and hippocampal neurogenesis [29,30]. CREB has a well-known role in brain neuroplasticity and long-term memory formation, and has been shown to be essential in the formation of spatial memory [31]. Additionally, accumulated Aβ can decrease BDNF and CREB proteins in brain tissue [32]. Treatment with the probiotic *L. mesenteroides* H40 alleviated the decrease in BDNF and CREB levels (Figure 6). CREB binds to BDNF promoter IV and is phosphorylated in response to BDNF-TrkB signaling, recruiting CBP to activate the transcription of BDNF promoter IV [33]. Lab4b, *L. plantarum* IS-1506, and heat-killed *L. lactis* KC24 increased BDNF mRNA expression [5,34,35].

Excessive reactive oxygen species (ROS) cause neurodegenerative diseases, and scopolamine reduces antioxidant capacity [36,37]. The antioxidant effects of probiotic *L. mesenteroides* H40 are shown in Figure 7. Superoxide dismutase (SOD) is the first detoxification enzyme involved in ROS production. It decomposes two molecules of the superoxide anion (*O_2_) into hydrogen peroxide and molecular oxygen (O_2_), rendering the potentially harmful superoxide anions less harmful. Catalase can decompose hydrogen peroxide into oxygen and water in peroxisomes. GSH-Px decompose hydrogen peroxide into water in the mitochondria. Since these enzymes were increased by the probiotic *L. mesenteroides* H40 treatment, these results suggest that it prevents cellular damage due to oxidative stress.

In humans, cognitive impairment was influenced by social circumstances (education, economic status, drink, etc.) [38,39,40]. However, in animal model can imitate the disease progression than the human.

## 5. Conclusions

Treatment with the probiotic *L. mesenteroides* H40 attenuated cognitive disorders in a scopolamine-induced cognitive disorder mouse model. The behavioral deficits were further improved by probiotic *L. mesenteroides* H40. The neuroprotective effects of probiotic *L. mesenteroides* H40 were demonstrated by controlling neuroinflammation, neurotransmitters, synaptic plasticity, Aβ accumulation, and antioxidant effects in brain tissue. These results imply that the probiotic *L. mesenteroides* H40 is an effective natural neuroprotective agent that can be used as a preventive functional food.

## Data Availability

Data is contained within the article.

## Abbreviations

The following abbreviations are used in this manuscript:

iNOS	Inducible nitric oxide synthase
COX-2	Cyclooxygenase-2
BDNF	Brain-derived neurotrophic factor
CREB	cAMP response element-binding protein
AD	Alzheimer’s disease
mAChRs	Muscarinic acetylcholine receptors
LPS	Lipopolysaccharide
PBS	Phosphate buffered saline
ELISA	Enzyme-linked immunosorbent assay
ACh	Acetylcholine
AChE	Acetylcholinesterase
ChAT	Choline acetyltransferase
SOD	Superoxide dismutase
ANOVA	One-way analysis of variance
GSH-Px	Glutathione peroxidase
